# Oral Myco- and Bacteriobiota and Yeast Infections in Mechanically Ventilated COVID-19 Patients

**DOI:** 10.3390/microorganisms11061442

**Published:** 2023-05-30

**Authors:** Iwona Gregorczyk-Maga, Michal Kania, Beata Sulik-Tyszka, Magdalena Namysł, Anna Sepioło, Dorota Romaniszyn, Estera Jachowicz-Matczak, Jadwiga Wójkowska-Mach

**Affiliations:** 1 Institute of Dentistry, Faculty of Medicine, Jagiellonian University Medical College, ul. Montelupich 4, 31-155 Kraków, Poland; 2 Doctoral School of Medicine and Health Sciences, Jagiellonian University Medical College, ul. św. Anny 12, 31-008 Kraków, Poland; 3 Chair of Metabolic Diseases, Faculty of Medicine, Jagiellonian University Medical College, ul. Jakubowskiego 2, 30-688 Kraków, Poland; 4 Department of Dental Microbiology, Medical University of Warsaw, Banacha 1A, 02-097 Warsaw, Poland; 5 Department of Microbiology, University Hospital in Krakow, Jakubowskiego Street 2, 30-688 Kraków, Poland; 6 Department of Microbiology, Faculty of Medicine, Jagiellonian University Medical College, ul. Czysta 18, 31-121 Kraków, Poland

**Keywords:** fungal microbiota, fungal community, yeasts, *Candida*, candidemia, mycoses, yeast infections, candidiasis, invasive candidiasis

## Abstract

Critically ill COVID-19 patients requiring mechanical ventilation in the intensive care unit are at risk of developing invasive candidiasis. In this study we aimed to (1) characterize oral cultivable mycobiota of mechanically ventilated adult COVID-19 patients in an ICU setting by sampling four distinct oral niches in two fixed time points with regards to oral health status, (2) investigate *Candida* spp. infections in this population, and (3) compare oral mycobiota with selected bacteriobiota strains during the observation in the ICU. We recruited 56 adult COVID-19 patients who qualified for mechanical ventilation. Patients received either standard or extended oral care procedures with tooth brushing. Oral samples were taken first within 36 h and after 7 days of intubation. Yeast-like fungi were identified by MALDI/TOF mass spectrometry. Yeast infection cases were retrospectively analyzed. *Candida* spp. in oral sampling was identified in 80.4% and 75.7%, *C. albicans* in 57.1% and 61.1%, and *non*-*albicans Candida* species in 48.2% and 47.2% patients at baseline and follow-up, respectively. There were no differences in the overall CFU counts of *Candida* spp. species and individual *Candida* species in oral samples, both at baseline and follow-up. At baseline, a higher prevalence of *Candida* spp. was associated with a higher identification rate of *Lactobacillus* spp. (64.4% vs. 27.3%, *p* = 0.041). At follow-up, there was a borderline lower prevalence of *Candida* spp. in patients with *Lactobacillus* spp. identified (57.1% vs. 87.0%, *p* = 0.057). The incidence rate of candidiasis was 5.4% and the incidence density was 3.1/1000 pds. In conclusion, *non-albicans Candida* species in oral samples were identified in nearly half of patients. Oral health was moderately impaired. A high incidence of yeast infections, including invasive cases, in patients hospitalized in the ICU due to COVID-19 and requiring mechanical ventilation was noted. Severe COVID-19 and disease-specific interventions within the ICU possibly played a major role promoting *Candida* spp. infections.

## 1. Introduction

*Candida* spp. is one of the most important components of human microbiota [1]. Of various fungi, mainly the yeast-like *Candida* genus, especially *Candida albicans*, plays an import role in oral cavity colonization [1]. The prevalence of *C. albicans* colonization has been previously reported to range between 15 and 30% [1]. In critically ill patients, the isolation rate of *Candida* spp. can be as high as 50% [2]. In immunocompromised patients, these commensal species can colonize the lower respiratory system and form pathological biofilms on the mucosal surfaces, which has previously been associated with longer duration of mechanical ventilation, increased risk of ventilator associated pneumonia, increased length of intensive care unit (ICU) stay, and higher mortality [3,4]. In this population, *Candida* spp. can cause opportunistic yeast infections [4]. Previous studies suggested multiple bidirectional interactions between *Candida* spp. and oral bacteriota [4,5,6]. Oral bacteria can facilitate yeast infections by promoting the expression of virulence genes in *C. albicans*. Reversely, *C. albicans* may change the antibiotic resistance patterns of pathogenic bacteria when coexisting in biofilms [7,8]. Recent studies showed that patients with periodontitis have an increased risk of complications in the course of COVID-19 infection and that COVID-19 can exacerbate periodontal disease [9]. Treatment of periodontal disease, including photodynamic therapy adjunctive to standard antimicrobial treatment and mechanical methods of scaling and root planing, may be treated as a preventive measure against potential exacerbation of COVID-19 [9,10].

COVID-19 is caused by the SARS-CoV-2 and causes mild to moderate respiratory illness. In individuals with comorbidities or with compromised immune systems, the risk of severe forms of COVID-19, including acute respiratory distress syndrome (ARDS), is higher [11]. Such critically ill COVID-19 patients, when admitted to the intensive care unit (ICU) and requiring mechanical ventilation, are more likely to develop healthcare-associated infections [12,13]. Cases of both bacterial and fungal infections were reported, with studies focusing on multidrug resistant bacterial strains, mucormycosis, and aspergillosis [12,14,15,16]. As *Candida* spp. infections in ventilated COVID-19 patients are still under-researched [3], in this study, we intended to provide new evidence in this matter. By implementing individual sampling niches twice—immediately after the initiation of mechanical ventilation and 7 days afterwards—we tried to provide a thorough characterization of oral microbiota and their changes during the hospitalization in the ICU.

In this study, we aimed to (1) characterize oral cultivable mycobiota of mechanically ventilated adult COVID-19 patients in an ICU setting by sampling four distinsct oral niches in two fixed time points with regards to oral health status, (2) investigate *Candida* spp. infections in this population, and (3) compare oral mycobiota with selected bacteriobiota strains during the observation in the ICU.

## 2. Materials and Methods

Adult patients admitted to the University Hospital in Krakow, Poland between 1 September 2021 and 31 January 2022,were offered the opportunity to participate in the study and asked for the signed consent form on admission to the hospital. During hospitalization, 56 of them qualified for intubation and were hospitalized in the temporary intensive care unit (ICU) for COVID-19 patients.

The inclusion criteria were as follows:

1. SARS-CoV-2 infection confirmed by real-time reverse transcriptase-polymerase chain reaction (RT-PCR) assay of nasal and pharyngeal swabs upon hospital admission;
2. Signed consent to participate in the study;
3. Patients were admitted to the ICU;
4. ntubation due to COVID-19-related pneumonia and acute respiratory distress syndrome (ARDS) within 36 h preceding study procedures.

Demographic and clinical data were gathered from the hospital electronic medical records, including but not limited to age, sex, date of COVID-19 diagnosis, admission to the hospital and ICU, date of intubation, selected comorbidities, and pre- and postintubation treatment, including systemic steroids and antibiotics. SOFA (Sequential Organ Failure Assessment) [17] was calculated at baseline and follow-up. Selected baseline and maximal laboratory results were also extracted. Information on bacterial and yeast infection during the hospitalization was recorded.

### 2.1. Oral Cavity Sampling Methods

During the observation, patients received two types of oral care—the standard procedure that included cleaning, moisturizing of oral cavity and suction of excess fluid or the extended procedure with additional tooth brushing. The detailed description of the intervention and its effects on oral bacteriobiota has been published elsewhere [13].

The oral cavity health status was inspected and the presence of symptoms of oral candidiasis was recorded. Oral health was assessed using a modified Beck Oral Assessment Scale (BOAS) consisting of 5 subscales, namely assessment of lips, mucosa and gingiva, tongue, teeth, and saliva. Oral samples from four oral habitats (buccal mucosa, tongue, buccal dental surface and gingival pocket) were taken two times, first within 36 h of intubation (baseline) and again after 7 days of intubation (follow-up). Every sample was taken by a trained dentist.

ESwabs™ (COPAN-invented flocked swab with 1 mL of liquid amine in a plastic, screw cap tube, COPAN Diagnostics, Muriera, CA, USA) were used for sampling mucosal surfaces of the posterior part of the dorsum of the tongue and buccae. Tooth Cleanic KerrHawe—KWX-OP-SZ-011 (KerrHawe SA, Biggio, Switzerland) was used to collect the dental plaque from the buccal dental surface side. After the collection, the brush was placed in 1 mL of Liquid Amies in a plastic screw cap tube. Gingival crevicular fluid (GCF) samples were collected with three pieces of PerioPaper Strips (Oraflow, Smithtown, NY, USA), designed to absorb 0–1.2 microliters of fluid. The strips were placed in the gingival pocket for 30–45 s, and then in ESwab tubes (COPAN Diagnostics, Muriera, CA, USA). To prevent a contamination of GCF by saliva, sterile gauze was used to dry the tooth surfaces and remove excess saliva from the mucosae.

The collected samples were immediately delivered to the Chair of Microbiology of Jagiellonian University Medical College. The samples were inoculated for variety of microbiological media to identification bacteria and fungi. The samples were inoculated by the dilution method (dilutions −1 to −6) or qualitative culture method (swabs only). For assessment of fungi growth, Chromagar Candida (Graso, Starogard Gdański, Poland), Sabouraud Agar (Biomaxima, Lublin, Poland) were used. Sabouraud agar and Chromagar Candida were aerobically incubated at 28 °C for 48 h.

The bacterial cultures and results were described in detail earlier [13,16]. After incubation, colonies were counted, reported, and assessed phenotypically. Results being presented as colony forming units (CFU) per mL (CFU/mL). The microorganisms were identified by MALDI TOF MS mass spectrometry (Vitek MS Home bioMérieux, with the V3 version of the database).

In this study, the data concerning yeast species identification and CFU counts from all four sampled oral sites were merged as we aimed to characterize the general status of oral mycobiota.

### 2.2. Diagnosis of Yeast Infections

Associated healthcare-associated infections (HAI) were identified according to definitions of the Healthcare-Associated Infections Surveillance Network (HAI-Net), European Centre for Disease Prevention and Control (protocol version 4.3), which concerns the ICU, taking into account the Guideline for the Management of Candidiasis of the Infectious Diseases Society of America concerning the diagnosis of candidiasis [18,19].

For the microbiological diagnosing of HAIs, clinical samples including blood, blood obtained from the catheter, tracheal or bronchial secretions, and urine obtained via freshly inserted bladder catheter were collected. Only the first isolate from each patient was selected for microbiological analysis, excluding subsequent cultures from the same patient and infection case. Mycological and bacteriological cultures were conducted in parallel for each material. Blood (arterial, venous, collected by central venous cathether [CVC]) was incubated in a BACT/ALERT**^®^** VIRTUO**^®^** system (Biomerieux, Marcy-l’Étoile, France). The clinical simples (blood from the positive media) were seeded on Sabouraud glucose agar + gentamicin + chloramphenicol (Thermo Scientific, Waltham, MA, USA) media and incubated at 37 °C (blood), at 35 °C (urine) or at 25 °C and 35 °C simultaneously (lower respiratory tract materials). Interpretation of growth and the titer of non-bronchoscopic lavage and urine cultures was based on ECDC and ASM methodology [19,20]. No additional tests were performed in patients enrolled in the study with positive culture of *Candida* spp., e.g., detection of *Candida* antigen and/or anti-*Candida* antibody in serum or in broncho-alveolar lavage (BAL). Due to severe acute respiratory distress, the BAL was not feasible.

### 2.3. Ethics Statement

The study and its protocol were approved by the Jagiellonian University Bioethics Committee, decision numbers 1072.6120.333.2020, 7 December 2020 and 1072.6120.353.2020, 16 December 2020. Written informed consent was obtained from each subject prior to participation. Trial registration number: 1072.6120.333.2020.

### 2.4. Statistical Analysis

The PS Imago Pro ver. 7.0 was used for all statistical analyses. The normality of the continuous variable distribution was assessed using the Shapiro‒Wilk test. Differences between groups were analyzed with Student’s *t* test or nonparametric test (Mann–Whitney U test) when appropriate. Paired data were analyzed using the Wilcoxon test. Continuous variables are presented as the arithmetic mean ($\overset{-}{x}$) ± standard deviation (SD) or as the median with interquartile range (IQR) when the data were not normally distributed. The distribution of categorical variables was described as counts and percentages. Statistical testing was completed to compare categorical variables using an independent sample chi-squared test or Fisher’s exact test when appropriate and dependent samples with McNemar’s test. We measured the strength association by odds ratio (OR) and 95% confidence intervals (CI). Statistical inference was set at *p* < 0.05.

## 3. Results

### 3.1. Demographic and Clinical Characteristics

The study population included 56 adult patients admitted to an ICU ward who required mechanical ventilation due to COVID-19-related pneumonia. The mean age of the participants was 66.5 ± 12.7 years, and there were 24 (42.9%) females. The population was obese with a mean BMI of 31.9 ± 5.8. Sixteen (28.6%) were admitted directly from the emergency ward, and forty (71.4%) were transferred from another hospital ward. The mean time from COVID-19 diagnosis to intubation was 6.95 ± 6.62 days. On admission to ICU, the median BOAS was 12 (IQR 10–14), and on the follow-up was 11 (IQR 9–14), showing moderate dysfunction of oral health.

Pre-ICU, systemic steroid therapy was used in 76.9%, antibiotics in 63.5%, and antifungal agents in 14.3% of patients. There were no differences in the SOFA score at baseline and follow-up. The full pre-ICU characteristics of the study population have been previously described elsewhere [21]. In the ICU, steroids, antibiotics and antifungal agents were used in 85.7%, 55.4%, and 10.6% of patients, respectively. The overall mortality in this cohort was 76.8% throughout the whole hospitalization period, with 7-day mortality of 33.9%. The clinical characteristics of the study participants are presented in Table 1.

### 3.2. Oral Cultivable Mycobiota Composition

There were no signs of oral thrush either at baseline or follow-up in the studied population. No white plaques or reddish atrophic areas were observed. At baseline, Candida spp. was identified in 80.4% patients. The most common was *C. albicans,* identified in 57.1%. *Non-albicans Candida species* were present in 48.2% of patients with *C. dubliniensis* in 17.9%, followed by *C. glabrata* in 16.1% and *C. kefyr* in 14.3% of patients, with the remaining species present in singular cases.

Candida spp. were identified in 75.7% of patients at follow-up. C. albicans was present in 61.1%, and non-albicans Candida species was present in 47.2%. C. dubliniensis were identified in 13.5%, C. kefyr in 13.5%, and C. glabrata in 18.9% of patients. The remaining species were identified rarely. There were no significant differences in the frequency of identification of Candida spp. between the baseline and follow-up sampling. There were no differences in the mycobiota composition between the four prespecified sampling sites, either at baseline or follow-up. The oral mycobiota composition is presented in Table 2.

**Figure 1:**
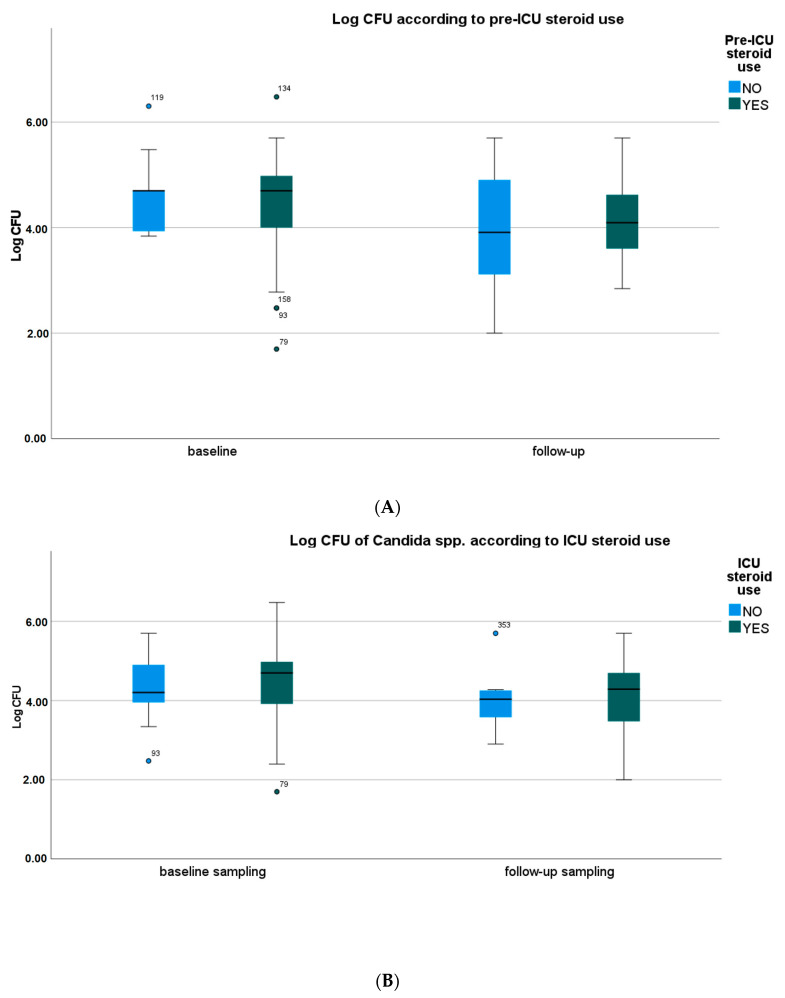

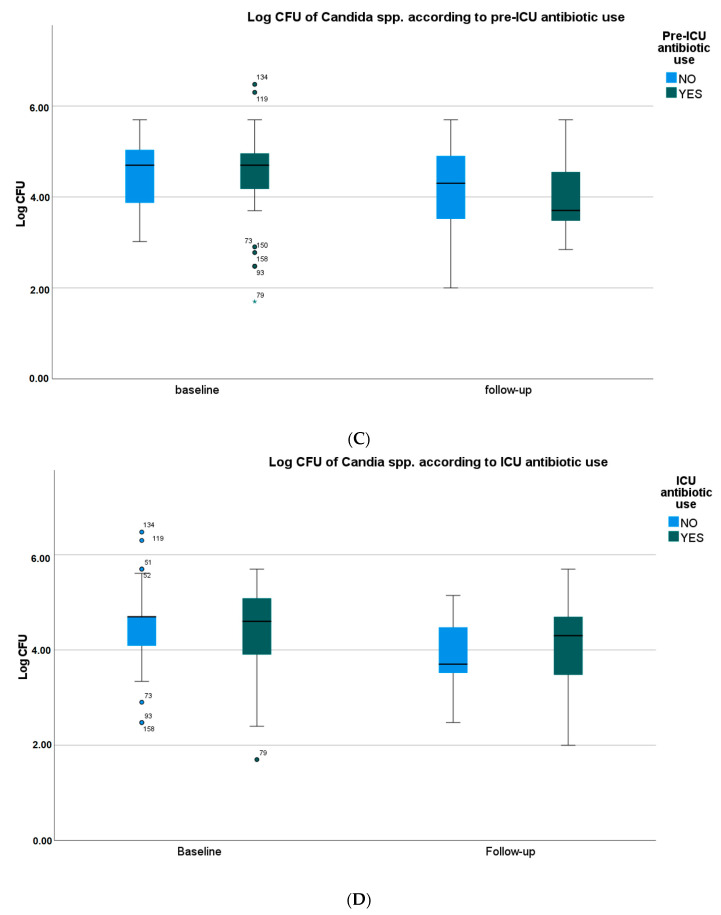
Yeast species density according to the use of selected drugs. (**A**) Yeast species density according to the pre-ICU use of systemic steroids. (**B**) Yeast species density according to the ICU use of systemic steroids. (**C**) Yeast species density according to the pre-ICU use of antibiotics. (**D**) Yeast species density according to the ICU use of antibiotics. CFU—colony-forming unit. Medians, IQRs, errors, outliers (dot), and extreme values (asterisk) are presented.

Pre-ICU and in-ICU antifungal agent use, history of diabetes, oral health status, type of oral care procedure, and SOFA score did not significantly affect the frequency of identification and CFU counts of *Candida* spp. in oral samples. However, the use of steroids and antibiotics, both pre-ICU and in ICU, showed a trend towards higher CFU counts of *Candida* spp. in baseline and follow-up samples (Figure 1A–D, Tables S2–S8).

Numerically, CFU counts of Candida spp. strains decreased between the baseline and follow-up sampling. However, there were no significant differences in the overall CFU counts of all Candida spp. species, C. albicans and non-albicans Candida species, and individual Candida species, both at baseline and follow-up (Table 3).

### 3.3. Comparison of Mycobiota with Selected Bacteriobiota Strains

**Figure 2:**
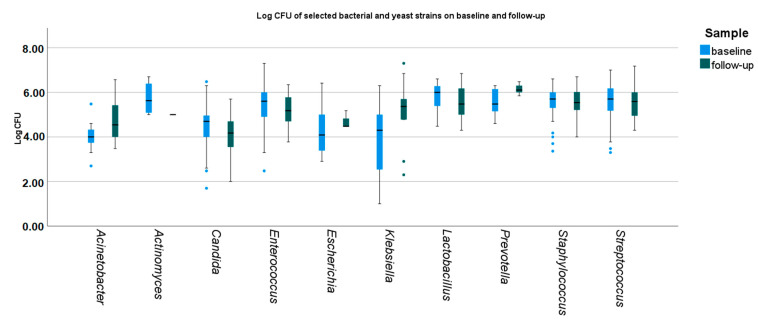
Baseline and follow-up density of selected bacterial and yeast strains. Medians, IQRs, errors, and outliers (dots) are presented. CFU—colony-forming unit.

Between the baseline and follow-up oral sampling, a decrease in the overall number of bacteria and fungi species from all sites was observed (median 6 vs. 4, *p* = 0.005). There were no significant differences in the overall CFU counts of bacterial and *Candida* spp. strains (Figure 2, Table S1).

**Figure 3:**
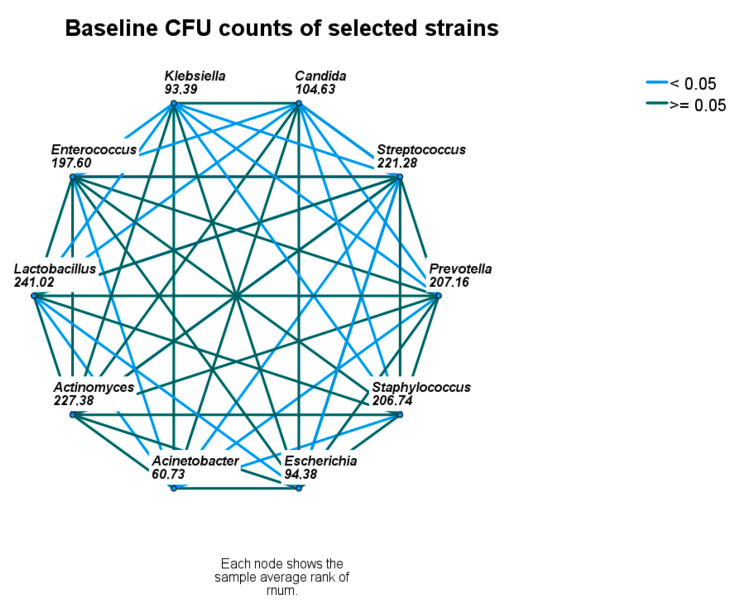
Baseline comparison of microbial densities. Nodes connected with blue lines differ significantly. CFU—colony-forming unit.

At *baseline*, the CFU counts of identified microbial strains differed significantly, namely *Streptococcus* spp., *Staphyloccus* spp., and *Lactobacillus* were higher, and *Candida* spp., *Escherichia coli*, *A. baumanii*, and *K. pneumoniae* were lower than the remaining strains (Figure 2 and Figure 3).

**Figure 4:**
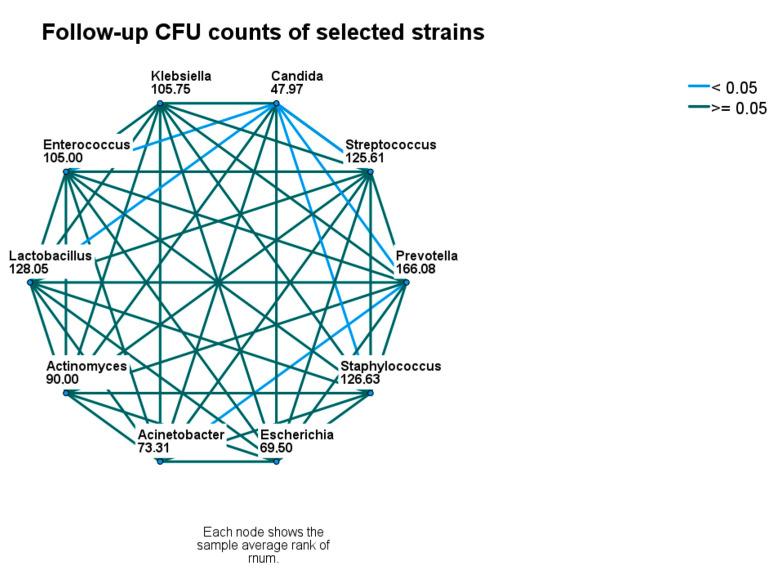
Follow-up comparison of microbial densities. Nodes connected with blue lines differ significantly. CFU—colony-forming unit.

At follow-up, significant differences between the CFU counts of identified microbial strains were less common. CFU counts of *Candida* spp. were still lower than *Enterecoccus* spp., *Lactobacillus* spp., *Prevotella* spp., and *Streptococcus* spp. and *Staphylococcus* spp. The CFU counts of *Prevotella* spp. remained higher than *Candida* spp. and *A. baumanii*. (Figure 2 and Figure 4).

Considering differences between identification rates of *Candida* spp. and selected bacterial strains in oral samples from all sampled oral niches, at baseline, higher prevalence of *Candida* spp. was associated with a higher identification rate of *Lactobacillus* spp. (64.4% vs. 27.3%, *p* = 0.041). Conversely, at follow-up, there was a lower prevalence of *Candida* spp. in patients with *Lactobacillus* spp. identified, but it was borderline insignificant (57.1% vs. 87.0%, *p* = 0.057) [11]. There were no associations with the identification rate of *A. baumannii*, *K. pneumoniae, E. faecalis,* and *P. aeruginosa* and the presence of *Candida* spp strains.

### 3.4. Yeast Infections

*Candida* spp. infections were recognized in 7 patients:

- 2 cases of microbiologically confirmed CVC-related bloodstream infection (*C. albicans* and *C. tropicalis*) without quantitative CVC blood sample or quantitative or semi-quantitative CVC culture;
- 1 case of primary bloodstream infection, *C. glabrata;*
- 2 cases of mixed bacterial-fungal symptomatic urinary tract infection:
  ○ *C. glabrata* and *A. baumannii*
  ○ *C. albicans* and Enterobacter cloeacae;
- 2 putative cases of mixed bacterial–fungal symptomatic urinary tract infection, in both cases the urine specimens were taken from a Foley catheter:
  ○ *C. albicans* and Enterobacter cloeacae
  ○ *C. parapsilosis* and Enterococcus faecium;
- 1 putative case of pneumonia—the mixed culture of *C. lusitaniae*, *C. inconspicua*, *C. tropicalis,* and *C. albicans* with 104 CFU/mL for each were obtained with non-bronchoscopic lavage.

The incidence rate of invasive yeast infections was 5.4%, with the incidence density rate of 3.1/1000 patient days (pds). The incidence rate of all true and putative yeast infections was 12.5%, with the incidence density rate of 7.3/1000 pds.

In the two cases of CVC-related bloodstream infection caused by *C. albicans* and *C. tropicalis*, the same species were identified in both the samples from the oral cavity. In the case of primary bloodstream infection caused by *C. glabrata*, the strain of *C. glabrata* was also identified in the oral samples.

## 4. Discussion

The presence of *Candida* spp. in oral samples, with no signs of thrush or inflammatory lesions on the oral mucosa, indicates that only the oral colonization with *Candida* spp. was observed in the studied population. Previous studies revealed that *Candida* spp. comprise oral mycobiota, being present in 30–80% of healthy individuals [22]. Only a small portion of patients with oral *Candida* spp. colonization present signs of candidiasis [23]. Immunocompromised patients and those hospitalized in the ICU have a higher risk of developing oral candidiasis due to its adhesion ability, antigenic variation, increased production of hydrolytic enzymes, and immunomodulatory activity [24]. In our study, in patients with severe COVID-19 admitted to the ICU and requiring mechanical ventilation, there were no cases of oral candidiasis, but the oral colonization with *Candida* spp. was common. *C. albicans* was identified most frequently, but a remarkably high prevalence of *non-albicans Candida* species in nearly half of the patients was also observed. Of *non-albicans Candida* species, *C. dublinensis* dominated, followed by *C. glabrata* and *C. kefyr*. To explore any potential shifts during the initial period after ventilation, the oral cavity was sampled once more. The colonization rate remained high in the second sampling 7 days postintubation regardless of antimicrobial drugs and steroid treatment, oral health status, and oral care procedures. Similar to our findings, *C. albicans* was reported to be the most common species, with marked presence of *C. dublinensis, Candida glabrata, Candida parapsilosis, Candida krusei, Candida tropicalis,* and *Candida pseudotropicalis* [22,25]. The colonization of the oral cavity with *Candida* species is not per se pathological, but as an opportunistic microorganism, it may predispose selected individuals, especially the immune incompetent, to invasive infections [24]. Other factors leading to higher risk invasive candidiasis development include altered local oral mucosal environment, antibiotic or steroid administration, coinfections, age, diabetes, smoking, history of surgical procedures within the oral cavity, or improper oral hygiene [26,27,28,29,30].

Patients in critical condition and hospitalized in the ICU are at even higher risk of invasive candidiasis. It was reported that approx. 30% of the patients in the ICU setting show fungal colonization (defined as *Candida* spp. growth in at least one sample other than blood, such as urinary culture, are tracheal aspirates) without clinical findings of infection. *C. albicans* was the most common species [31,32]. Severe COVID-19 alone is a life-threatening infection, with nearly 50% mortality reported [33,34]. As our data showed, the total mortality in this cohort reached 76.8% throughout the whole hospitalization period, with 7-day mortality of 33.9%. Candidemia in an ICU setting has been proven to show high mortality rates exceeding 50% [35,36,37]. Severe COVID-19 associated with candidemia in patients hospitalized in the ICU has the potential to form an extremely dangerous combination.

Many factors have been proposed to predispose for the development of invasive candidiasis in COVID-19 patients. First, the severity of COVID-19 infection possibly played a major role affecting the immune response to infection, enhancing gut microbiota translocation, promoting *Candida* spp. colonization and qualitative shifts towards higher identification rate of *non-albicans Candida* species [38]. Previous studies showed significant alarming dysbiosis of oral bacteriota in such populations [18], with high rates of healthcare-acquired infections of *Acinetobacter baumannii, Enterococcus faecalis, Escherichia coli,* and *Klebsiella pneumoniae* etiology [13]. Poor oral health status, high frequency of oral colonization by potentially pathogenic bacteria and early postintubation dysbiosis may also play a significant role in the development of candidiasis [13]. The use of corticosteroids was previously reported as the common risk factor for invasive candidiasis due to a complex dysregulation of host immune response to infections [39]. This was also confirmed for COVID-19 patients, in whom, as compared to historical cohorts, the consumption of corticosteroids was even higher, increasing this risk even further [38,40,41]. Numerous studies reported on the widespread use of broad-spectrum antibiotics in COVID-19 patients [33,39]. This led to the dysregulation of normal microbiome in multiple environmental niches. As a result, *Candida* spp. colonization in the oral cavity and gut was promoted [30,42]. One study also addressed this to high frequency of the use of instrumentation in COVID-19 patients in ICUs, including central venous catheters, arterial lines, and urine bladder catheters [40]. In this study, we identified two cases of *Candida* spp. bloodstream infections related to central venous catheter (CVC). CVC-related infections are common in the ICU setting, comprising ca. 75% of all ICU bloodstream healthcare-associated infections [43,44]. The risk of candidemia and eventually invasive candidemia is increased in patients with CVC [45]. Additionally, factors, such as age, severe hepatic failure, severity of disease, SOFA score, and septic shock, are unfavorably influencing the prognosis of candidiasis acquired in an ICU [35,42]. Finally, organizational factors may influence the incidence of *Candida* spp. CVC-related bloodstream infections, with various reported rates for medical vs. surgical ICUs or differing antibiotic or antifungal prophylaxis policies [46].

The reported incidence of putative yeast infections and invasive yeast infections in our study was higher than in data from previously reported historical cohorts. In a large pre-COVID-19-pandemic report from multiple sites in Europe, the cumulative incidence of invasive candidiasis was 7.07 episodes per 1000 ICU admissions [46]. Studies covering the COVID-19 pandemic consistently reported an increase in the rate of invasive candidiasis, with its rates ranging from 0.7 to 23.5% [40]. An increase in the candidemia rate (up to 10-fold) during the COVID-19 pandemic was also reported [32,38,47,48,49,50,51]. In our cohort, we did not find any statistically significant predisposing factors leading to development of candidiasis, probably due to small number of included patients. Similar observations were made by other study groups [47,49].

The etiologies of yeast infections in our cohort encompassed *C. albicans* and *non-albicans Candida* species. *C. albicans* has been reported to be the most frequent cause of invasive candidiasis and candidemia [32,38,49,51], which, however, was not confirmed in our study. Similar results are reported by Papadimitriou-Olivgeris et al., who showed that *non-albicans Candida* species predominated both before and during the pandemic period, with *C. parapsilosis* being the most common [52].

In patients that developed candidemia of *C. albicans, C tropicalis,* and *C. glabrata etiology*, the strains *of C. albicans, C tropicalis,* and *C. glabrata* were identified in samples from the oral cavity. However, we did not perform a molecular genotype analysis of oral and blood *Candida* spp. strains.

In one putative case of pneumonia, the suspicion of the case of infection was based on the non-BAL material and as such should be treated with caution. According to the literature, pneumonia caused by *Candida* spp. is exceptional in non-neutropenic patients. The collection of BAL samples is a reference method in the diagnostic process with the parallel use of cultivate and serological methods, supplemented by information from chest imaging and serological tests [53]. UTIs are rarely caused by fungi. The risk factors of candiduria include diabetes, neoplasm, bladder catheterization, the use of wide spectrum antibiotics, immune suppressive therapy, and surgical procedures. Candiduria may be the only sign of candidemia, and when observed during invasive candidiasis, it may be associated with higher mortality [54].

We noted a shift from the high prevalence of both *Candida* spp. and *Lactobacillus* spp. at baseline to divergent proportions at follow-up. We suspect that the presence of *Lactobacillus* spp. at baseline was due to the routine administration of probiotics pre-ICU, according to the local infection prevention standards. In the ICU, however, the probiotics were discontinued. As the hospitalization progressed, the rate of *Lactobacillus* spp. dropped, and the rate of *Candida* spp. identification remained stable. There are limited reports on *Lactobacillus* spp. in displaying antifungal activities. It is assumed that it can produce substances with anticandidal action, contributing to lower prevalence of *Candida* spp. Still, the specifics of such antifungal mechanisms remain unexplored [5]. Future studies should investigate the impact of postbiotics and parabiotics as regulators of oral cavity homeostasis as they have shown promising results considering oral health and better outcomes of periodontal therapy [55,56,57,58].

Ensuring the safety of medical staff performing procedures in such a high-risk environment is crucial. As a previous review reported, minimally invasive oral treatments can reduce bacteremia and selected periodontal indices, advantaging both the staff and patients [55]. In the case of any pandemic situation, this is even more important, as the reduction of aerosol bacterial load is a major step towards ensuring safer work environment and less patients complications [59].

Our study has some strengths. As *Candida* spp. infections in ventilated COVID-19 patients are still under-researched, in this study we intended to provide new evidence in this matter. There have been no attempts to investigate the gingival pocket as the source of clinically significant samples of oral microbiota in mechanically ventilated COVID-19 patients. In most cases, oral swabs or saliva samples were tested. By sampling microbiologically diverse individual oral niches during the observation period, we were able to acquire a thorough characterization of oral bacterio- and mycobiota. Moreover, by sampling the oral cavity twice, immediately after the initiation of mechanical ventilation and 7 days afterwards, to our knowledge, for the first time, we were able explore the relationship between the oral mycobiota and selected oral bacterial species in regard to dynamic changes of oral health status in this cohort of patients.

Our study has several limitations. It was relatively small, single-centered, and of retrospective design. Since only patients with COVID-19 were treated in the investigated ICU, there was no control group with non-COVID-19 patients. In this study we used traditional methods for identification of microorganisms with additional MALDI-TOF for species classification. Moreover, the interpretation of yeast cultures obtained via Foley catheter or non-bronchoalveolar lavage should be performed with caution. Detection of *Candida* antigen and/or anti-*Candida* antibody in serum or in BAL were not utilized in this study. The pathogenicity of mixed bacterio-fungal biofilms may not be clear. Similarly, the differentiation between the colonization vs. clinically significant *Candida* spp. infection of the respiratory tract is problematic at times. Finally, we did not genotype the strains of *Candida* spp. isolated from oral samples and from infection cases.

## 5. Conclusions

In conclusion, we observed shifts towards a higher identification rate of *non-albicans Candida* species and divergent proportions of *Candida* spp. and *Lactobacillus* spp. in oral samples between the baseline and follow-up in mechanically ventilated adult COVID-19 patients in an ICU setting. Oral health was moderately impaired in this population. A high incidence of yeast infections, including invasive cases, was noted. Severe COVID-19 and disease-specific interventions within the ICU possibly played a major role promoting *Candida* spp. infections in our study population.

## Figures and Tables

**Table 1:** Clinical characteristics of study participants.

Characterisitcs	
Age [years]	66.5 (12.7)
Female [n (%)]	24 (42.9%)
BMI [kg/m^2^]	31.9 (5.8)
Baseline BOAS, sum score	12 (10–14)
Follow-up BOAS, sum score	11 (9–14)
Baseline SOFA score	13 (11–14)
Follow-up SOFA score	12 (9.8–13.3)
Steroid therapy before intubation [n (%)]	40 (76.9%)
Systemic antibiotic before intubation [n (%)]	33 (63.5%)
Systemic antifungal agents before intubation [n (%)]	6 (11.5%)
Systemic steroid therapy after intubation [n (%)]	48 (85.7%)
Systemic antibiotic after intubation [n (%)]	31 (55.4%)
Systemic antifungal after before intubation [n (%)]	8 (14.3%)

Data are presented as the means (SDs), medians (Q1–Q3), or n [%]; BOAS—Beck Oral Assessment Scale; SOFA—Sequential Organ Failure Assessment; NS—not significant.

**Table 2:** List of all identified Candia species in oral samples &.

	Baseline, N = 56		Follow-Up, N = 37 *		
Species					*p*
*Candida* spp.	45	80.4%	28	75.7%	NS
*Candida albicans*	32	57.1%	22	61.1%	NS
*Non-albicans Candida* spp.	27	48.2%	17	47.2%	NS
*Candida dubliniensis*	10	17.9%	5	13.5%	NS
*Candida glabrata*	9	16.1%	7	18.9%	NS
*Candida inconspicua*	3	5.4%	2	5.4%	NS
*Candida kefyr*	8	14.3%	5	13.5%	NS
*Candida krusei*	1	1.8%	0	-	-
*Candida lusitaniae*	2	3, 6%	0	-	-
*Candida parapsilosis*	1	1.8%	2	5.4%	NS
*Candida spherica*	1	1.8%	0	-	-
*Candida tropicalis*	2	3, 6%	3	8.1%	NS

Numbers of patients with strains identified [N (%)] are presented; &—samples from all prespecified oral niches; * number of patients who survived until the follow-up; NS—not significant.

**Table 3:** Oral mycobiota composition—quantitative analysis.

	Baseline, N = 56	Follow-Up, N = 37 *	*p*
CFU/mL of *Candida* spp. from oral samples &	5.0 × 10^4^ (8.1 × 10^3^ − 8.8 × 10^4^)	1.2 × 10^4^ (3.0 × 10^3^ − 5.0 × 10^4^)	NS
CFU/mL of *Candida albicans* from oral samples &	5.0 × 10^4^ (1.0 × 10^4^ − 9.1 × 10^4^)	5.1 × 10^3^ (1.5 × 10^4^ − 4.8 × 10^4^)	NS
CFU/mL of *non-albicans Candida* spp. from oral samples &	5.0 × 10^4^ (8.6 × 10^3^ − 3.0 × 10^5^)	2.0 × 10^4^ (6.5 × 10^3^ − 5.0 × 10^4^)	NS

Data are presented as the means (SDs), medians (Q1–Q3) or N [%]; CFU—colony-forming unit; * number of patients who survived until the follow-up; &—samples from all prespecified oral niches; NS—not significant.

## Data Availability

The data presented in this study are available on request from the corresponding author.

## Supplementary Materials

The following supporting information can be downloaded at: https://www.mdpi.com/article/10.3390/microorganisms11061442/s1.
