# Supplementary material for: Oral Myco- and Bacteriobiota and Yeast Infections in Mechanically Ventilated COVID-19 Patients
Source: Microorganisms. 2023 May 30;11(6):1442. doi: 10.3390/microorganisms11061442 (PMC10301763; doi:10.3390/microorganisms11061442)
Supplement: Supplementary file 1 [file microorganisms-11-01442-s001.zip › microorganisms-2400405-supplementary.pdf]

Table S1. Microbial strain density according to sampling.			
Baseline CFU/ml from oral samples	Baseline	Follow-up	P value
Acinetobacter baumannii	1.0E+04 (2.3E+03 – 2.6E+04)	3.5E+04 (8.8E+04 – 2.8E+05)	NS
Candida spp.	2.3E+04 (5.0E+3 – 7.9+E04)	1.2E+04 (3.0E+3 – 5.0E+04)	NS
Enterococcus spp.	4.0E+05 (7.8E+04 – 1.0E+06)	1.5E+05 (4.5E+04 – 6.0E+05)	NS
Escherichia coli	1.3E+04 (2.3E+03 – 3.1E+05)	3.0E+04 (3.0E+04 – NA)	NS
Klebsiella pneumoniae	2.0E+04 (3.0E+02 – 1.0E+05)	2.4E+05 (4.5E+04 – 2.1E+06)	NS
Lactobacillus spp.	1.0E+06 (2.0E+05 – 2.0E+06)	3.0E+05 (1.0E+05 – 1.6E+06)	NS
Prevotella spp.	3.0E+05 (1.0E+05 – 1.5E+06)	1.3E+06 (9.3E+05 – 2.3E+06)	NS
Streptococcus spp.	5.0E+05 (1.5E+05 – 1.5E+06)	4.0E+05 (8.5E+04 – 1.0E+-6)	NS
Staphylococcus spp.	5.0E+05 (2.0E+05 – 1.0E+06)	3.5E+05 (1.5E+05 – 1.0E+06)	NS
data are presented mean (SD), median (Q1-Q3), CFU – colony forming unit			

Table S2. SOFA score in patients according to Candida spp. identification in oral samples						
	Candida spp. present vs. non present at baseline	*Candida albicans* present vs. non present at baseline	*Candida* non *albicans* present vs. non present at baseline	*Candida* spp. present vs. non present at follow-up	*Candida albicans* present vs. non present at follow-up	*Candida* non *albicans* present vs. non present at follow-up
SOFA score, baseline	NS	NS	NS	NS	NS	NS
SOFA score, follow-up	NS	NS	NS	NS	NS	NS
SOFA - Sequential Organ Failure Assessment, NS – not significant						

Table S3. Baseline and follow-up characteristics by oral health status category.						
	Baseline			Follow-up		
Characteristics	BOAS 0-10	BOAS 11-20	p value	BOAS 0-10	BOAS 11-20	p value
Percentage of patients with selected genera/species in oral samples at baseline						
Candida spp.	84.6%	80.6%	NS	92.9%	73.7%	NS
Candida albicans	53.8%	63.9%	NS	71.4%	42.1%	NS
Candida non-albicans	53.8	41.7%	NS	50.0%	47.4%	NS
Percentage of patients with selected genera/species in oral samples at follow-up						
Candida spp.	70.0%	79.2%	NS	78.6%	73.7%	NS
Candida albicans	50.0%	73.9%	NS	69.2%	63.2%	NS
Candida non-albicans	50.0%	66.7%	NS	53.8%	36.8%	NS
CFU/ml from oral samples from all oral sites at baseline						
All *Candida* spp.	2.0E+04 (3.8E+03-5.0E+04)	1.4E+04 (2.2E+03-5.0E+04)	NS	8.6E+03 (3.0E+03-5.0E+04)	3.0E+04 (8.0E+03-5.0E+04)	NS
CFU/ml from oral samples from all oral sites at follow-up						
All *Candida* spp.	4.5E+04 (1.5E+04-2.1E+05)	5.0E+04 (7.8E+3-1.3E+05)	NS	1.9E+04 (4.5E+03-5.1E+04)	5.0E+04 (4.1E+04-3.0E+05)	NS
data are presented as the means (SDs), medians (Q1-Q3) or N [%]; BOAS – Beck Oral Assessment Scale, NS – not significant						

Table S4. Baseline and follow-up characteristics by history of diabetes.						
	Baseline			Follow-up		
Characteristics	+	-	p value	+	-	p value
Percentage of patients with selected genera/species in oral samples						
Candida spp.	85.0%	77.8%	NS	80.0%	72.7%	NS
Candida albicans	65.0%	52.8%	NS	71.4%	54.5%	NS
Candida non-albicans	40.0%	52.8%	NS	42.9%	50.0%	NS
CFU/ml from oral samples from all oral sites						
All *Candida* spp.	1.9E+04 (4.0E+03-5.0E+04)	1.8E+04 (1.5E+03-4.0E+04)	NS	4.0E+04 (8.6E+03-7.3E+04)	5.0E+04 (4.1E+03-4.0E+05)	NS
data are presented as the means (SDs), medians (Q1-Q3) or N [%], NS – not significant						

Table S5. Baseline and follow-up characteristics by procedure performed.						
	Baseline			Follow-up		
Characteristics	Standard procedure	Extended procedure	p value	Standard procedure	Extended procedure	p value
Percentage of patients with selected genera/species in oral samples						
Candida spp.	88.0%	74.2%	NS	88.9%	63.2%	NS
Candida albicans	68.0%	48.4%	NS	77.8%	44.4%	NS
Candida non-albicans	52.0%	45.2%	NS	55.6%	38.9%	NS
CFU/ml from oral samples from all oral sites						
All *Candida* spp.	1.8+E04 (3.6E+03-5.0E+04)	1.5E+04 (3.1E+03-6.5E+04)	NS	5.0E+04 (5.1E+03-1.5E+05)	4.0E+04 (8.2E+03-1.3E+05)	NS
data are presented as the means (SDs), medians (Q1-Q3) or N [%], NS – not significant						

Table S6. Baseline and follow-up characteristics by antifungal agents use.						
	pre-ICU antifungal agents use			in-ICU antifungal agents use		
Characteristics	-	+	p value	-	+	p value
Percentage of patients with selected genera/species in oral samples at baseline						
Candida spp.	84.8%	50.0%	NS	79.2%	87.5%	NS
Candida albicans	60.9%	33.3%	NS	56.3%	62.5%	NS
Candida non-albicans	50.0%	50.0%	NS	43.8%	75.0%	NS
Percentage of patients with selected genera/species in oral samples at follow-up						
Candida spp.	82.8%	40.0%	NS	72.4%	87.5%	NS
Candida albicans	71.4%	33.3%	NS	60.7%	62.5%	NS
Candida non-albicans	46.4%	40.0%	NS	42.9%	62.5%	NS
CFU/ml from oral samples from all oral sites at baseline						
All *Candida* spp.	1.4E+04 (2.2E+03-5.0E+04)	4.3E+04 (9.8E+03-3.1E+05)	NS	1.7E+04 (2.0E+03-5.0E+04)	1.8E+04 (5.0E+03-8.0E+04)	NS
CFU/ml from oral samples from all oral sites at follow-up						
All *Candida* spp.	5.0E+04 (8.9E+03-1.4E+05)	1.1E+04 (1.4E+03-1.4E+05)	NS	5.0E+04 (5.0E+03-2.0E+05)	5.0E+04 (1.4E+04-9.0E+04)	NS
data are presented as the means (SDs), medians (Q1-Q3) or N [%], NS – not significant						

Table S7. Baseline and follow-up characteristics by antibiotic use.						
	pre-ICU antibiotic use.			in-ICU antibiotic use.		
Characteristics	-	+	p value	-	+	p value
Percentage of patients with selected genera/species in oral samples at baseline						
Candida spp.	78.9%	81.8%	NS	80.0%	80.6%	NS
Candida albicans	47.4%	63.6%	NS	64.0%	51.6%	NS
Candida non-albicans	52.6%	48.5%	NS	40.0%	54.8%	NS
Percentage of patients with selected genera/species in oral samples at follow-up						
Candida spp.	71.4%	80.0%	NS	80.0%	74.1%	NS
Candida albicans	64.3%	63.2%	NS	60.0%	61.5%	NS
Candida non-albicans	42.9%	47.4%	NS	40.0%	50.0%	NS
CFU/ml from oral samples from all oral sites at baseline						
All *Candida* spp.	8.0E+03 (2.0E+03-3.0E+04)	2.3E+04 (4.2E+03-5.0E+04)	NS	2.5E+04 (4.4E+03-5.0E+04)	1.4E+04 (2.0E+03-5.0E+04)	NS
CFU/ml from oral samples from all oral sites at follow-up						
All *Candida* spp.	5.0E+04 (3.0E+04-2.5E+05)	3.9E+04 (6.6E+03-6.6E+04)	NS	3.9E+04 (4.9E+03-5.0E+04)	5.0E+04 (9.6E+03-2.3E+05)	NS
data are presented as the means (SDs), medians (Q1-Q3) or N [%], NS – not significant						

Table S8. Baseline and follow-up characteristics by steroid use.						
	pre-ICU steroid use			in-ICU steroid use		
Characteristics	-	+	p value	-	+	p value
Percentage of patients with selected genera/species in oral samples at baseline						
Candida spp.	75.0%	82.5%	NS	87.5%	79.2%	NS
Candida albicans	41.7%	62.5%	NS	62.5%	56.3%	NS
Candida non-albicans	58.3%	47.5%	NS	75.0%	43.8%	NS
Percentage of patients with selected genera/species in oral samples at follow-up						
Candida spp.	62.5%	80.8%	NS	80.0%	75.0%	NS
Candida albicans	50.0%	68.0%	NS	60.0%	61.3%	NS
Candida non-albicans	50.0%	44.0%	NS	40.0%	48.4%	NS
CFU/ml from oral samples from all oral sites at baseline						
All *Candida* spp.	8.0E+03 (3.0E+02-5.0E+04)	1.9E+04 (4.1E+04-5.1E+04)	NS	1.1E+04 (2.2E+02-4.0E+04)	2.0E+04 (4.0E+03-5.0E+4)	NS
CFU/ml from oral samples from all oral sites at follow-up						
All *Candida* spp.	5.0E+04 (1.0E+04-1.9E+05)	4.6E+04 (8.9E+03-1.3E+05)	NS	1.9E+04 (1.0E+04-8.0E+04)	5.0E+04 (6.0E+03-1.5E+05)	NS
data are presented as the means (SDs), medians (Q1-Q3) or N [%], NS – not significant						
